# *Fance* deficiency inhibits primordial germ cell proliferation associated with transcription–replication conflicts accumulate and DNA repair defects

**DOI:** 10.1186/s13048-023-01252-9

**Published:** 2023-08-10

**Authors:** Zhixian Zhou, Huan Yin, Suye Suye, Zhen Ren, Lei Yan, Liye Shi, Chun Fu

**Affiliations:** Department of Obstetrics and Gynecology, The Second Xiangya Hospital of Central South University, Changsha, 410000 China

**Keywords:** Fance, Premature ovarian insufficiency, Primordial germ cell, DNA repair, DNA replication, Transcription–replication conflicts

## Abstract

Fanconi anemia (FA) gene mutations are critical components in the genetic etiology of premature ovarian insufficiency (POI). *Fance*^*−/−*^ mice detected meiotic arrest of primordial germ cells (PGCs) as early as embryonic day (E) 13.5 and exhibited decreased ovarian reserve after birth. However, the mechanism of *Fance* defect leading to dysgenesis of PGCs is unclear. We aimed to explore the effect of *Fance* defects on mitotic proliferation of PGCs. Combined with transcriptomic sequencing and validation, we examined the effect of *Fance* defects on cell cycle, transcription–replication conflicts (TRCs), and multiple DNA repair pathways in PGCs during active DNA replication at E11.5 and E12.5. Results showed *Fance* defects cause decreased numbers of PGCs during rapid mitosis at E11.5 and E12.5. Mitotic cell cycle progression of *Fance*^*−/−*^ PGCs was blocked at E11.5 and E12.5, shown by decreased cell proportions in S and G2 phases and increased cell proportions in M phase. RNA-seq suggested the mechanisms involved in DNA replication and repair. We found *Fance*^*−/−*^ PGCs accumulate TRCs during active DNA replication at E11.5 and E12.5. *Fance*^*−/−*^ PGCs down-regulate multiple DNA repair pathways at E11.5 and E12.5 including the FA pathway, homologous recombination (HR) pathway, and base excision repair (BER) pathway. In conclusion, *Fance* defect impaired the mitotic proliferation of PGCs leading to rapidly decreased numbers and abnormal cell cycle distribution. Proliferation inhibition of *Fance*^*−/−*^ PGCs was associated with accumulated TRCs and down-regulation of FA, HR, BER pathways. These provided a theoretical basis for identifying the inherited etiology and guiding potential fertility management for POI.

**Supplementary Information:**

The online version contains supplementary material available at 10.1186/s13048-023-01252-9.

## Introduction

Premature ovarian insufficiency (POI) effects approximately 1–5% of women under the age of 40, resulting in decreased ovarian reserve and even infertility, thus seriously damaging women’s reproductive health [1, 2]. Genetic factors account for approximately 20–25% of POI etiology, including chromosomal abnormalities, genetic polymorphisms, and monogenic mutations [3–5]. Fanconi anemia (FA) genes encode proteins involved in gonadal development, DNA replication and DNA repair, and many studies have reported that mutations (*FANCA, -C, -G, -L, -M*) cause POI. However, the frequency of FA gene mutations in POI patients is unclear. Studies have reported 15 FA gene deficient female mouse models show decreased ovarian reserve and low fertility [6]. Fanconi anemia complementation group E (FANCE) plays a core role in the FA pathway and is involved in the construction of FA core complexes (FANCA, -B, -C, -E, -F, -G, -L, -M, -T) and linking downstream FANCD2-FANCI monoubiquitylation complexes, DNA damage repair proteins (FANCD1, -J, -N, -O, -P, -Q, -R, -S, -U, -V, -W) and their coordinated FA-associated proteins [7, 8]. Previous studies by our group reported that *Fance*^*−/−*^ female mice showed reduced numbers and stagnated pachytene of primordial germ cells (PGCs) during meiosis starting as early as embryonic day (E) 13.5, and also showed reduced numbers of follicles at all levels in the ovary and reduced fertility during postnatal 1–6 weeks of age, which are similar to the human young female POI phenotype [9, 10]. However, the effect of *Fance* deficiency on PGCs development during rapid mitotic proliferation before E13.5 is unclear.

PGCs of embryonic mice proliferate rapidly during the mitotic period to establish adequate reproductive reserve. Faced with the high DNA replication pressure during the rapid proliferation phase, PGCs maintain genomic stability by relying critically on accurate DNA replication and repair, which are essential for cell survival [11, 12]. DNA replication is the central step in germ cell proliferation and is strictly regulated by the cell cycle [13, 14]. DNA replication stalls in the presence of various replication barriers blockages, which include transcription–replication conflicts (TRCs) and various DNA damages such as interstrand cross-links (ICLs), protein-DNA cross-links, single-strand breaks, double-strand breaks (DSBs), etc. [15–17]. Various DNA repair pathways work collaboratively to remove replication barriers such as DNA damage and provide precise DNA templates for DNA replication, contributing to successful DNA replication [18]. DNA replication and DNA repair are coordinated to maintain genomic stability and to promote rapid mitotic proliferation of PGCs for establishing adequate ovarian reserve. However, the effect of the *Fance* defect on the cellular activity of DNA replication and repair during mitotic proliferation of PGCs is not known.

We aimed to investigate the effect of *Fance* defect on cell development during the period of rapid mitotic proliferation of PGCs before E13.5 (E11.5-E12.5). Our study explored the changes in PGCs number and the distribution of mitotic cell cycles in *Fance*^*−/−*^ embryonic mice. We also used RNA sequencing (RNA-Seq) analysis and examined the changes in DNA replication, TRCs, and DNA repair pathways in PGCs of *Fance*^*−/−*^ embryonic mice. These studies are contributing to explaining the mechanism of *Fance* defect causing reduced ovarian reserve in mammals. In addition, it also provides the theoretical basis for identifying the inherited etiology and guiding potential fertility management for POI.

## Materials and methods

### Animals and genotype identification

All experiments were performed according to the approval guidelines of the Laboratory Animal Welfare and Ethical Committee of Central South University (IACUC Number: 2020sydw1041). The research group has previously constructed *Fance*^*−/−*^ mice of FVB/N genetic background [9]. Mice were housed in Specific pathogen Free conditions (20–22℃, 12 h light/dark cycle) at the Central South University’s Laboratory Animals Department. Embryonic tissues were genotyped by DNA extraction, PCR amplification, and agarose gel electrophoresis as previously reported (Figure S1) [19]. The PCR primer sequences used for genotyping are listed in Table S1.

### PGCs isolated by flow cytometry

The urogenital crest of E12.5 embryos was isolated, digested overnight at 4 °C with 0.25% Trypsin-EDTA, neutralized with 10% fetal bovine serum (FBS) inactivated with trypsin and centrifuged at 300RCF for 5 min. The cell suspension was resuspended in PE-conjugated anti-mouse/human CD15 (SSEA1) (BioLegend, 125,606) diluted in 2.5% FBS and incubated for 30 min at room temperature. Afterward, cells were resuspended in 2.5% FBS and run through a BD FACS Aria II cell sorter. The PGCs cell suspension obtained by Fluorescence activated Cell Sorting (FACS) showed strong fluorescence under fluorescence microscope. PGCs were centrifuged at 500 RCF and stored at -80 °C.

### PGCs transcriptome sequencing and analysis

As shown in the previous research of the research group, sequencing was performed using an Illumina Nova6000 instrument provided by Genergy Biotechnology Co. Ltd. (Shanghai, China) [20]. Raw data reads and quality control was processed by Skewer v0.2.2 and FastQC v0.11.5, and aligned by STAR to the mouse Ensembl genome with annotation. The screening threshold of differentially expressed genes (DEGs) was FDR < 0.05 and |log2 Fold Change|>1.5. Gene Ontology (GO) and Kyoto Encyclopedia of Genes and Genomes (KEGG) were used to perform functional and signaling pathway enrichment analysis. In GO analysis, we evaluated enriched biological processes (BPs), molecular functions (MFs), and cellular components (CCs).

### Immunofluorescence staining

Embryos of pregnant female mice at E11.5 and E12.5 were used for the study. Embryonic mouse tissues were fixed in 4% paraformaldehyde and paraffin-embedded to perform continuous tissue sectioning at a thickness of 4 μm. Tissue sections were dewaxed, dehydrated, washed in PBS, and then subjected to antigen repair in Tris-EDTA (pH = 8.0) solution at high temperature and pressure for 15 min. The samples were incubated with 10% goat serum containing 0.3% TritonX-100 for 1 h at room temperature to permeabilize and block the samples. Tissue sections were incubated with primary antibody dilution overnight at 4℃, washed three times with PBS, and incubated with secondary antibody dilution for 1 h at room temperature. After washing with PBS, the sections were closed with anti-quenching reagent containing DAPI and observed under fluorescence microscopy. Antibodies used in the study included: SSEA1 (ab16285), PARP3 (PA5-112641), NEIL2 (PA5-103829), BLM (PA5-27384), RAD51 (NB100-148), LIG1 (MA5-42920), PCNA (SC-56), DNA-RNA Hybrid [S9.6] (Kf-Ab01137-23.0), FANCD2 (NB100-182), Cyclin B1(Cell Signaling, 4138), Alexa Fluor®594 Goat Anti-Rabbit (ab150080), Alexa Fluor®488 Goat Anti-Mouse (ab150117), Alexa Fluor®594 Goat Anti- Mouse (ab150116).

### EdU incorporation assay

Pregnant mice were injected intraperitoneally with 100 mg/kg EdU solution for 1 h and then killed. The isolated embryonic tissues were fixed in 4% paraformaldehyde, embedded in paraffin, and then continuously sectioned to a thickness of 4 μm. The tissue sections were dewaxed, dehydrated, and in Tris-EDTA (pH = 8.0) solution. Tissue sections were washed with PBS and incubated with 2 mg/mL glycine solution for 15 min, permeabilized with 0.5% TritonX-100 for 15 min, and incubated with EdU staining solution for 30 min according to the manufacturer’s instructions. After washing with PBS, tissue sections were labeled with SSEA1 protein using the same method of immunofluorescence staining as described above, and the sections were blocked after DAPI staining.

### Statistical analysis

Statistical analysis of the data was performed using GraphPad Prism 9.5.1. Comparisons between the two groups were made using ANOVA and Student’s two-sample t-test. *P* < 0.05 was considered a statistically significant difference. ns *P* ≥ 0.05, *P<0.05, **P<0.01, ***P<0.001, ****P<0.0001.

## Results

### Reduced PGCs numbers in *Fance*^*−/−*^ mice during rapid proliferation period

**Fig. 1:**
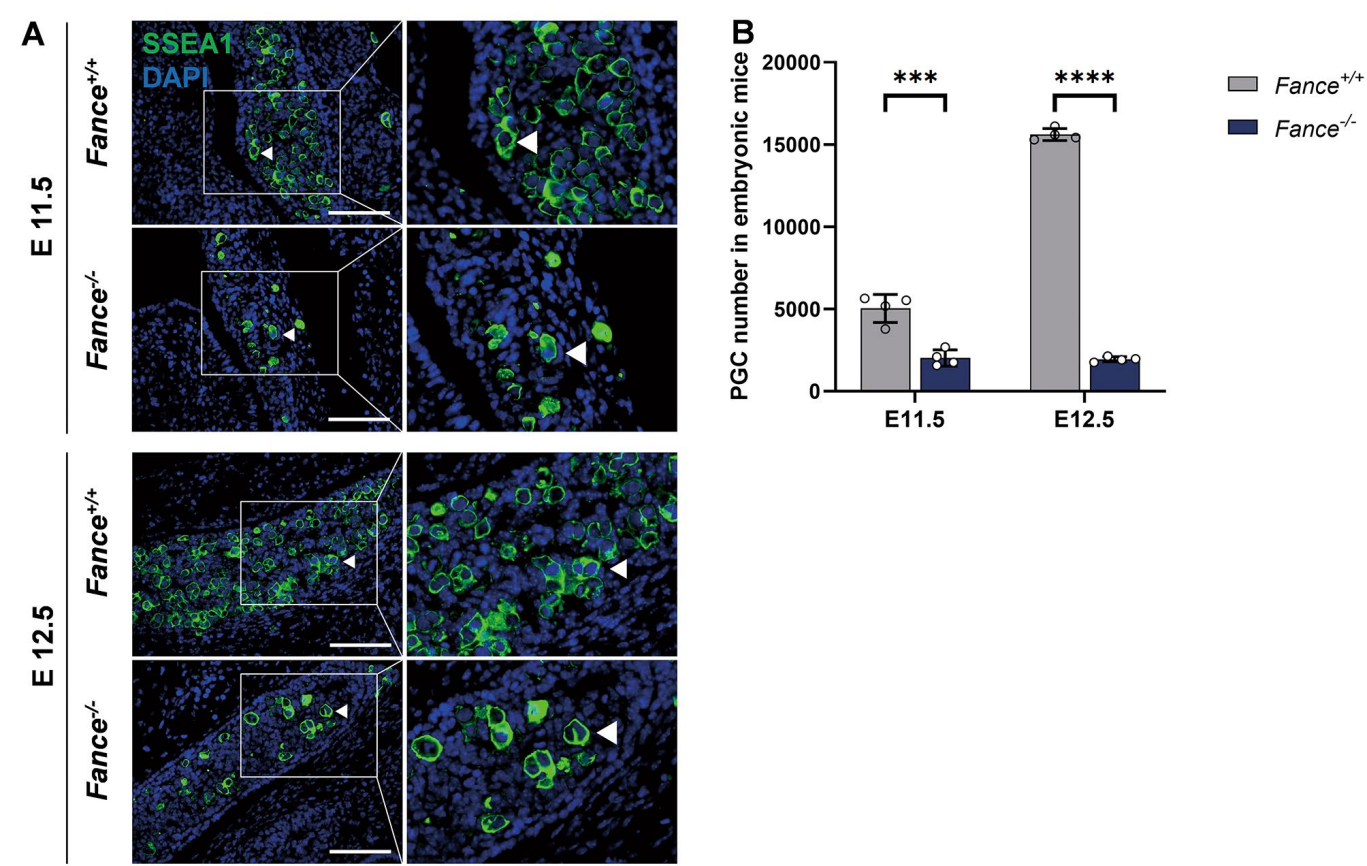
Reduced number of PGCs in *Fance* deficient mice during rapid mitosis. **(A)** Expression of the PGCs label protein SSEA1 was localized to the cell membrane in *Fance*^*+/+*^ and *Fance*^*−/−*^ female embryonic mice at E11.5-E12.5. **(B)** The PGCs number of *Fance*^*−/−*^ mice was significantly decreased than that of *Fance*^*+/+*^ mice. n = 4 embryos. All bars = 200 μm

PGCs before meiosis underwent continuous and rapid proliferation from E9.5 to E13.5, with a doubling time of 12.6 h for the number of PGCs [21]. Consistently, our results found an approximately 3-fold increase in the number of PGCs in mice of FVB/N genetic background from E11.5 to E12.5 during the mitotic period. Previous studies have shown that *Fance*^*−/−*^ mice exhibit significantly reduced PGCs numbers as early as E13.5, approximately 7.27% of the *Fance*^*+/+*^ mice [19]. We further found that the number of PGCs in *Fance*^*−/−*^ mice was significantly reduced during the proliferative phase with rapid mitosis. At E11.5, the number of PGCs in *Fance*^*−/−*^ mice was significantly reduced to about 40.19% of the *Fance*^*+/+*^ mice. With the continuous proliferation of PGCs in wild-type mice, the number of PGCs in *Fance*^*−/−*^ mice was about 12.41% of *Fance*^*+/+*^ mice at E12.5 (Fig. 1A-B). The results indicate that the *Fance* defect affects the rapid mitotic proliferation of PGCs during E11.5-E12.5. The results showed that mitotic proliferation in *Fance*^*−/−*^ mice was blocked during E11.5-E12.5, which presented a significant decrease in the number of PGCs compared to *Fance*^*+/+*^ mice.

### **Abnormal cell cycle distribution in*fance***^***−/−***^**PGCs during rapid proliferation period**

**Fig. 2:**
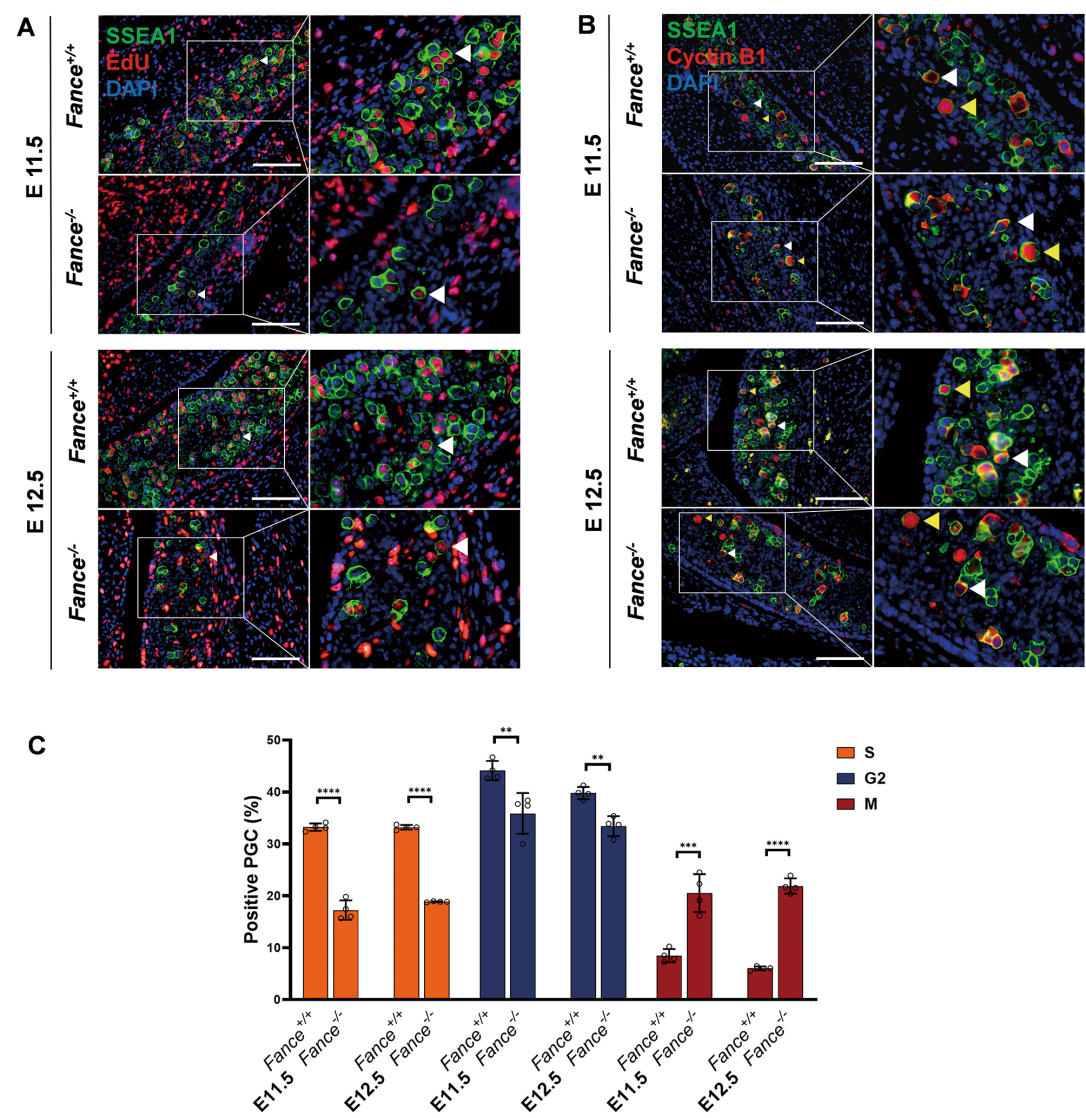
Abnormal cell cycle distribution in *Fance* deficient PGCs during rapid mitosis. **(A)** Representative images of co-localized staining of EdU and SSEA1 in *Fance*^*−/−*^ PGCs and *Fance*^*+/+*^ PGCs. **(B)** Representative images of co-localized staining of Cyclin B1 and SSEA1 in *Fance*^*−/−*^ PGCs and *Fance*^*+/+*^ PGCs. **(C)***Fance*^*−/−*^ mice had decreased proportion of PGCs in S and G2 phases and an increased proportion of PGCs in M phase compared to *Fance*^*+/+*^ mice (S, EdU positive; G2, Cyclin B1 positive in cytoplasm; M, Cyclin B1 positive in nucleus). n = 4 embryos. All bars = 200 μm

We next explored the cell cycle distribution of PGCs during continuous mitosis. EdU was injected into pregnant female mice at E11.5 and E12.5, embryos were isolated and PGCs stained to determine the amount of EdU infiltration and newly synthesized DNA. We combined EdU introgression and cyclin B1 staining to assess mitotic cell cycle progression in PGCs [22] (Fig. 2A-B). The results of the study found that *Fance* deficiency resulted in a significant reduction in the proportion of S-phase PGCs and the proportion of G2-phase PGCs, and a significant increase in the proportion of M-phase PGCs (Fig. 2C). The results indicate that *Fance* deficiency leads to cell cycle arrest and proliferation defects in PGCs. The results showed that the *Fance* defect resulted in significantly decreased proportion of PGCs in the DNA replication phase and significantly increased proportion of PGCs in the cell division phase.

### Transcriptome sequencing indicated *Fance*^*−/−*^ PGCs down-regulated replication and repair

**Fig. 3:**
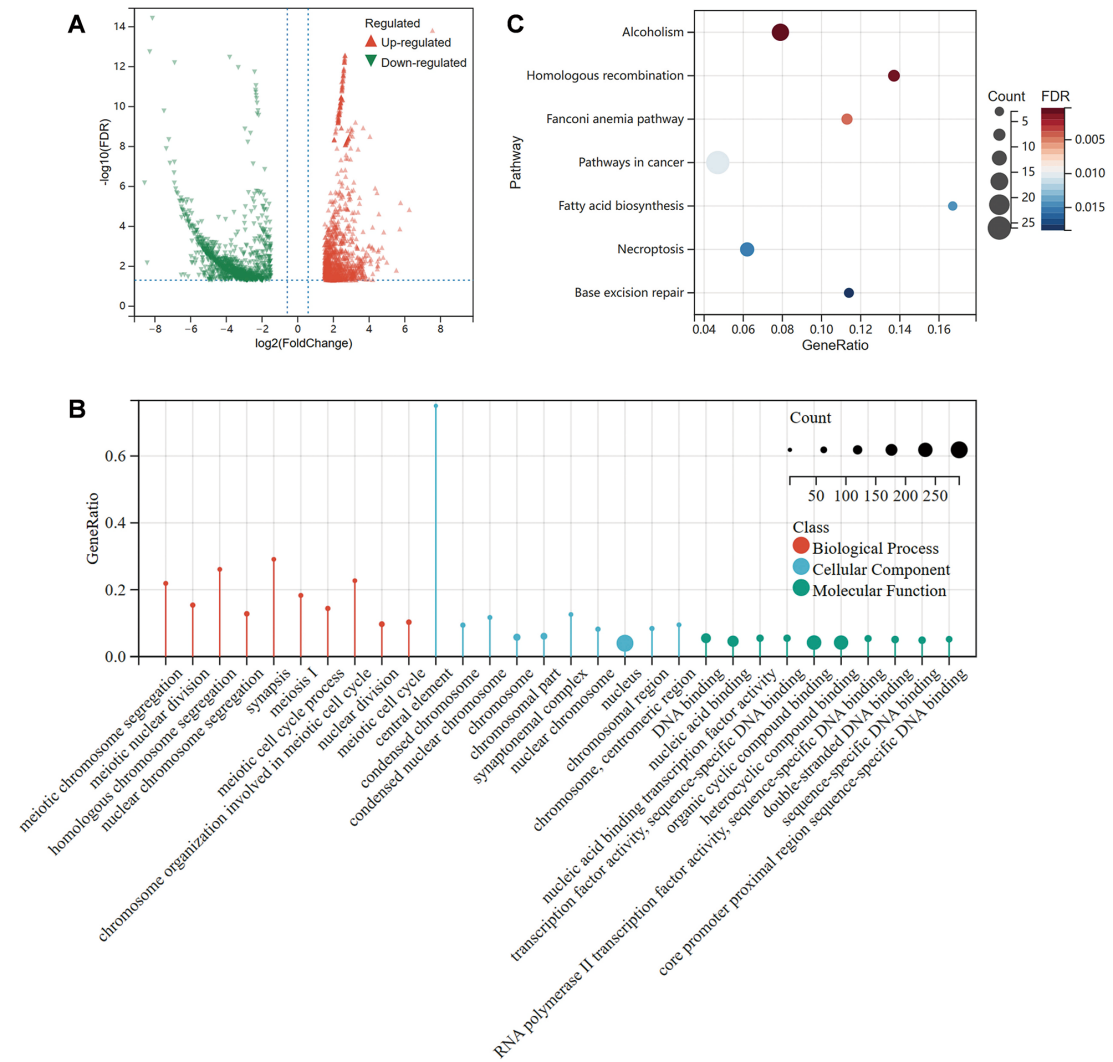
RNA-Seq analysis of transcriptome differences between *Fance*^*−/−*^ PGCs and *Fance*^*+/+*^ PGCs at E12.5. **(A)** Volcano plot of statistically significant DEGs at FDR < 0.05 and |log2 Fold Change|>1.5. **(B)** GO enrichment analysis of down-regulated DEGs. **(C)** KEGG enrichment analysis of down-regulated DEGs showed the top 7 enriched pathways

To further illustrate the mechanism involved in the blocked proliferation of PGCs in *Fance*^*−/−*^ mice, we sorted PGCs and then performed RNA-Seq to explore the whole-transcriptome differences in *Fance*^*−/−*^ PGCs compared to *Fance*^*+/+*^ PGCs at E12.5. A total of 2245 DEGs were identified, including 1019 up-regulated genes and 1226 down-regulated genes (Fig. 3A). We further used GO and KEGG enrichment analysis to explore the potential functions of the down-regulated DEGs more deeply from the biological function level. Results showed that down-regulated DEGs were mainly involved in DNA replication and repair in the nucleus. GO enrichment analysis showed down-regulation of DEGs significantly enriched items involving chromosome segregation, nuclear division (BP); nuclear chromosome (CC); DNA binding, and RNA transcription (MF) (Fig. 3B). KEGG enrichment analysis of down-regulated DEGs showed the top 7 enrichment pathways. Three of these pathways were classified in KEGG database as “Replication and repair”, including homologous recombination (HR), FA pathway, and base excision repair (BER) (Fig. 3C). These DNA repair mechanisms work together to repair DNA damage and play a key role in maintaining genome integrity [23, 24]. FANCE was an important member of the FA pathway and played an important role in the recruitment of multiple DNA repair proteins.

**Fig. 4:**
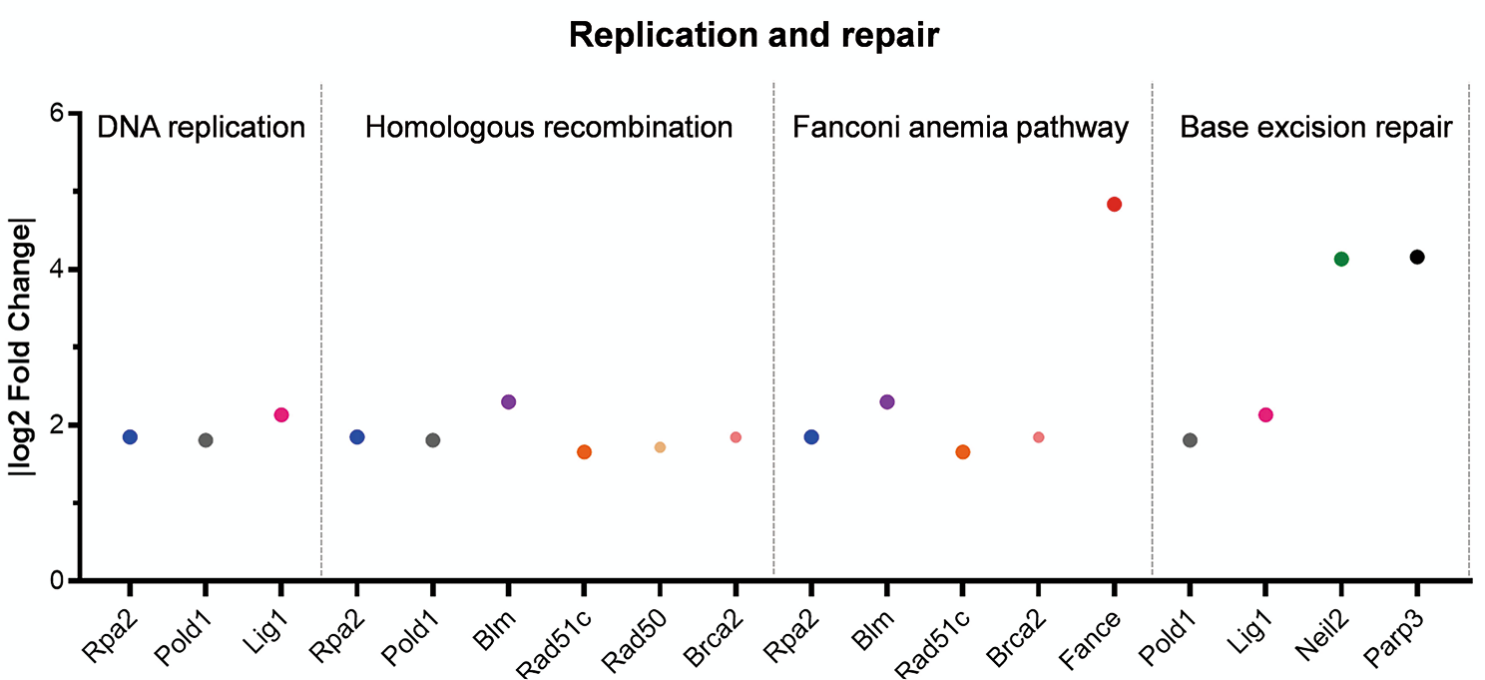
Compared to *Fance*^*+/+*^ PGCs, *Fance*^*−/−*^ PGCs down-regulated DEGs involved in replication and repair of KEGG classification. There shows the expression of DEGs involved in DNA replication, HR pathway, FA pathway and BER pathway by RNA-seq indication

Our subsequent investigation delved deeper into the underlying mechanism through which *Fance* defects impact the proliferation of PGCs. RNA-Seq results suggested that DNA replication and repair downregulation occurs in *Fance*^*−/−*^ PGCs. We found that the mRNA expression of several genes belonging to the “Replication and repair” classification in *Fance*^*−/−*^ PGCs was down-regulated based on RNA-Seq (Fig. 4). In addition, we further verified changes in DNA replication and several DNA repair pathways in *Fance*^*−/−*^ PGCs, compared to *Fance*^*+/+*^ PGCs.

### Replication-active *Fance*^*−/−*^ PGCs accumulate TRCs

**Fig. 5:**
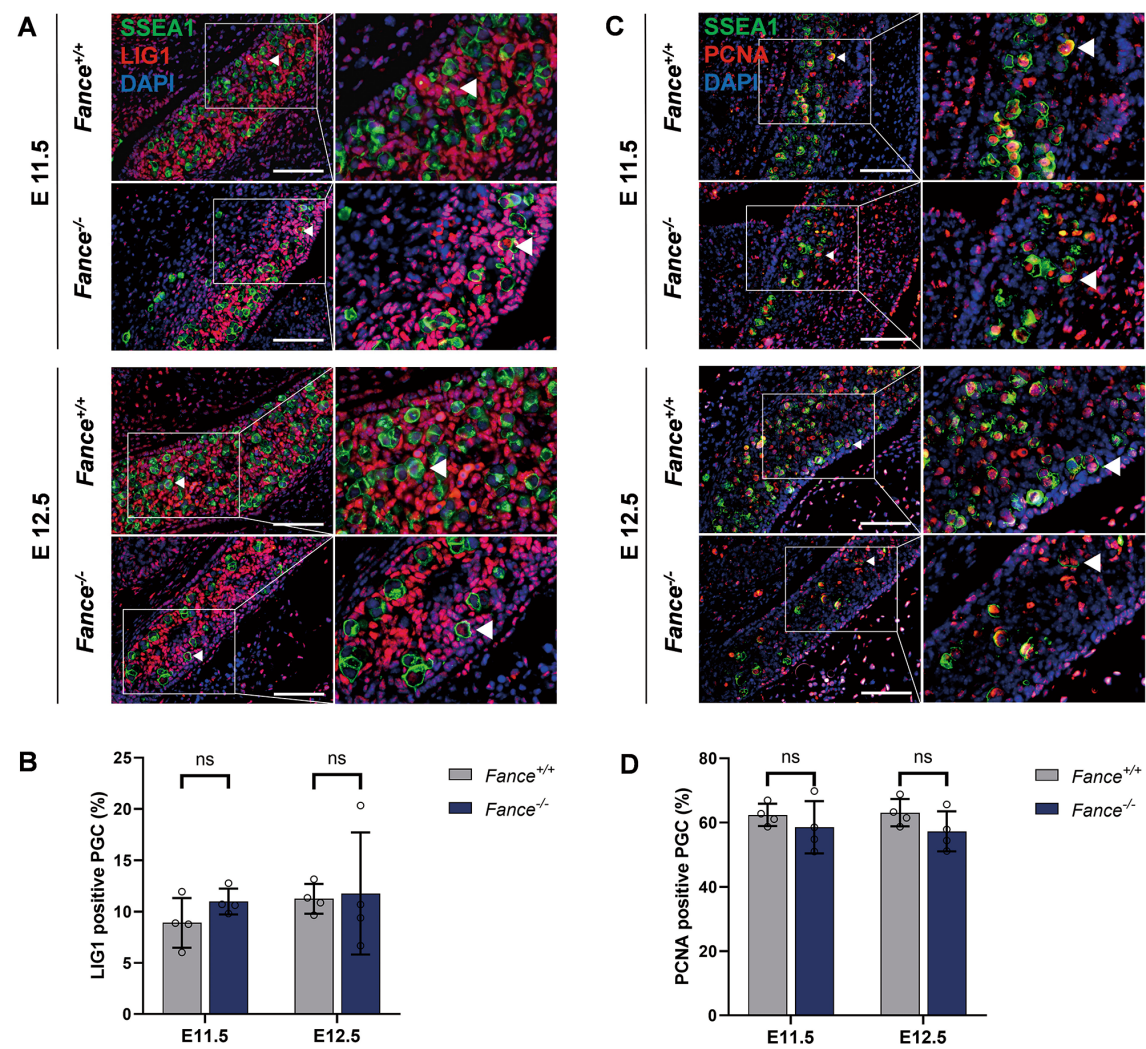
Expression of DNA replication proteins in *Fance*^*−/−*^ PGCs and *Fance*^*+/+*^ PGCs were not significantly different during rapid mitosis. **(A)** Representative images of co-localized staining of LIG1 and SSEA1 in *Fance*^*−/−*^ PGCs and *Fance*^*+/+*^ PGCs. **(B)** There was no significant difference in the proportion of LIG1 positive PGCs between *Fance*^*−/−*^ and *Fance*^*+/+*^ mice at E11.5 and E12.5. **(C)** Representative images of co-localized staining of PCNA and SSEA1 in *Fance*^*−/−*^ PGCs and *Fance*^*+/+*^ PGCs. **(D)** There was no significant difference in the proportion of PCNA positive PGCs between *Fance*^*−/−*^ and *Fance*^*+/+*^ mice at E11.5 and E12.5. n = 4 embryos. All bars = 200 μm

Our results showed significant reduction in the proportion of *Fance*^*−/−*^ PGCs in the DNA synthesis phase, including G2 and S phases. Thus, we evaluated the levels of DNA replication in PGCs. RNA-Seq results showed significant decrease in RNA expression of *Rpa2*, *Pold1*, *Lig1* in *Fance*^*−/−*^ PGCs at E12.5, these genes were involved in DNA replication (Fig. 4). We verified that there was no significant difference in the expression of DNA replication protein LIG1 in *Fance*^*−/−*^ PGCs and *Fance*^*+/+*^ PGCs at E11.5 and E12.5 (Fig. 5A-B). In addition, we detected there was no significant difference in the expression of the proliferating marker PCNA in *Fance*^*−/−*^ PGCs and *Fance*^*+/+*^ PGCs at E11.5 and E12.5 (Fig. 5C-D). Consistent with the previous results of our research group, there was no significant difference between the expression of another proliferative marker Ki67 in *Fance*^*−/−*^ PGCs and *Fance*^*+/+*^ PGCs during active mitosis [20]. These results indicate that *Fance* defects are not affecting proliferation associated proteins in PGCs during rapid mitosis. We consider that there were obstructions of replication in the PGCs that led to reduced DNA synthesis. Next, we examined changes in TRCs in *Fance*^*−/−*^ PGCs during active mitotic phase.

**Fig. 6:**
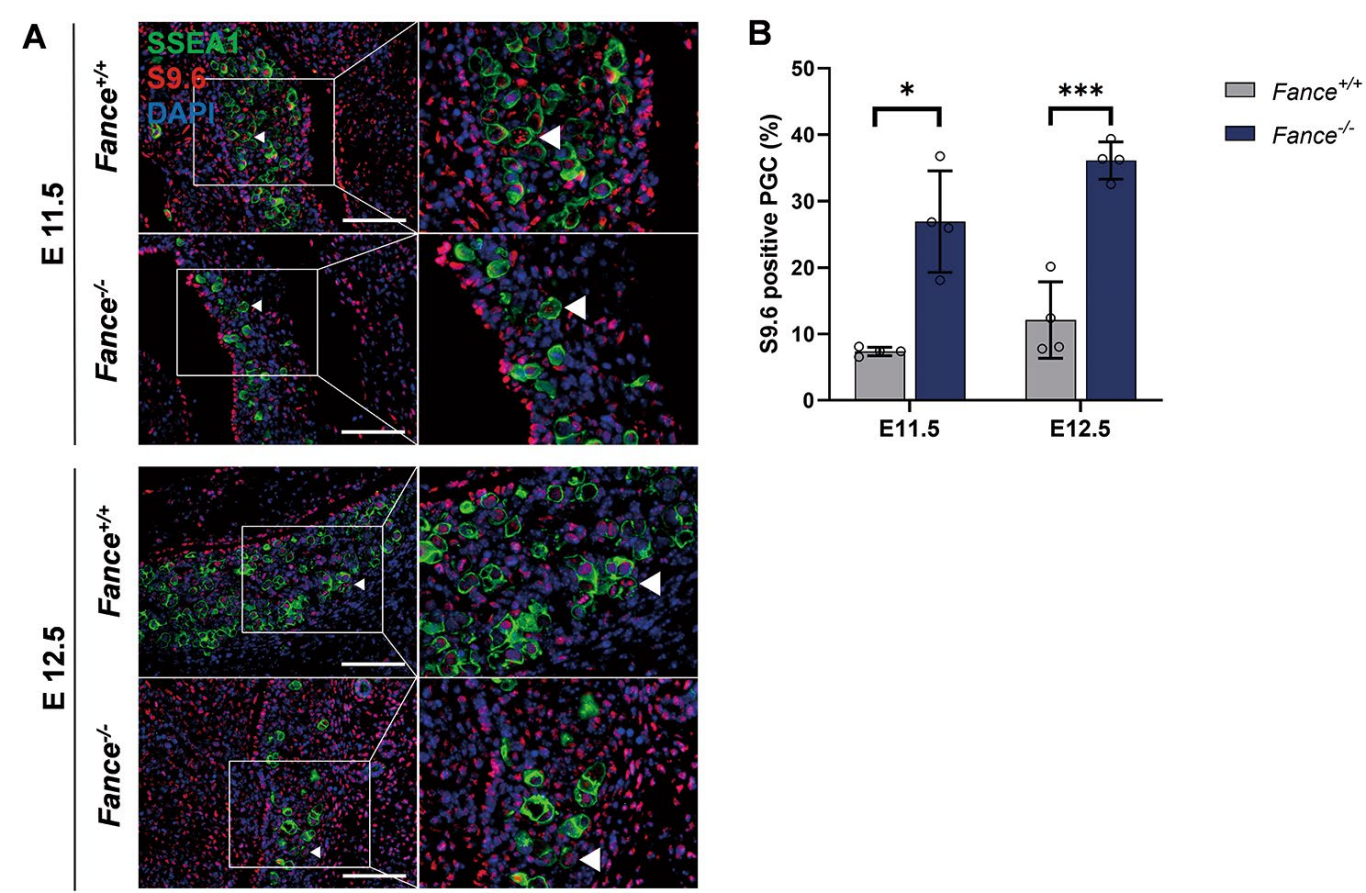
TRCs accumulate in *Fance* deficient PGCs during rapid mitosis. **(A)** Representative images of co-localized staining of R-loop (recognized by the S9.6 antibody) and SSEA1 in *Fance*^*−/−*^ PGCs and *Fance*^*+/+*^ PGCs. **(B)** The proportion of R-loop positive cells was significantly increased in *Fance*^*−/−*^ PGCs compared to *Fance*^*+/+*^ PGCs. n = 4 embryos. All bars = 200 μm

High levels of endogenous replication pressure exist in PGCs during active mitotic proliferation. DNA replication and transcription use the same DNA template, and both mechanisms inevitably encounter and generate TRCs, which serve as important sources of genomic instability [25, 26]. R loops are composed of DNA-RNA hybrids and translocated single-stranded DNA that can cause TRCs in a variety of organisms, disrupting DNA replication, causing DNA breaks and chromatin alterations [27–29]. We labeled the R loops with anti-S9.6 antibody and found significantly increased accumulation of R loops in *Fance*^*−/−*^ PGCs compared to *Fance*^*+/+*^ PGCs at E11.5 and E12.5 (Fig. 6A-B). The results suggested that *Fance* defects lead to the accumulation of R loops in the PGCs during rapid mitosis, resulting in TRCs, inducing high levels of endogenous replication pressure and blocking DNA replication in PGCs.

### ***Fance***^***−/−***^**PGCs down-regulated FA pathway**

**Fig. 7:**
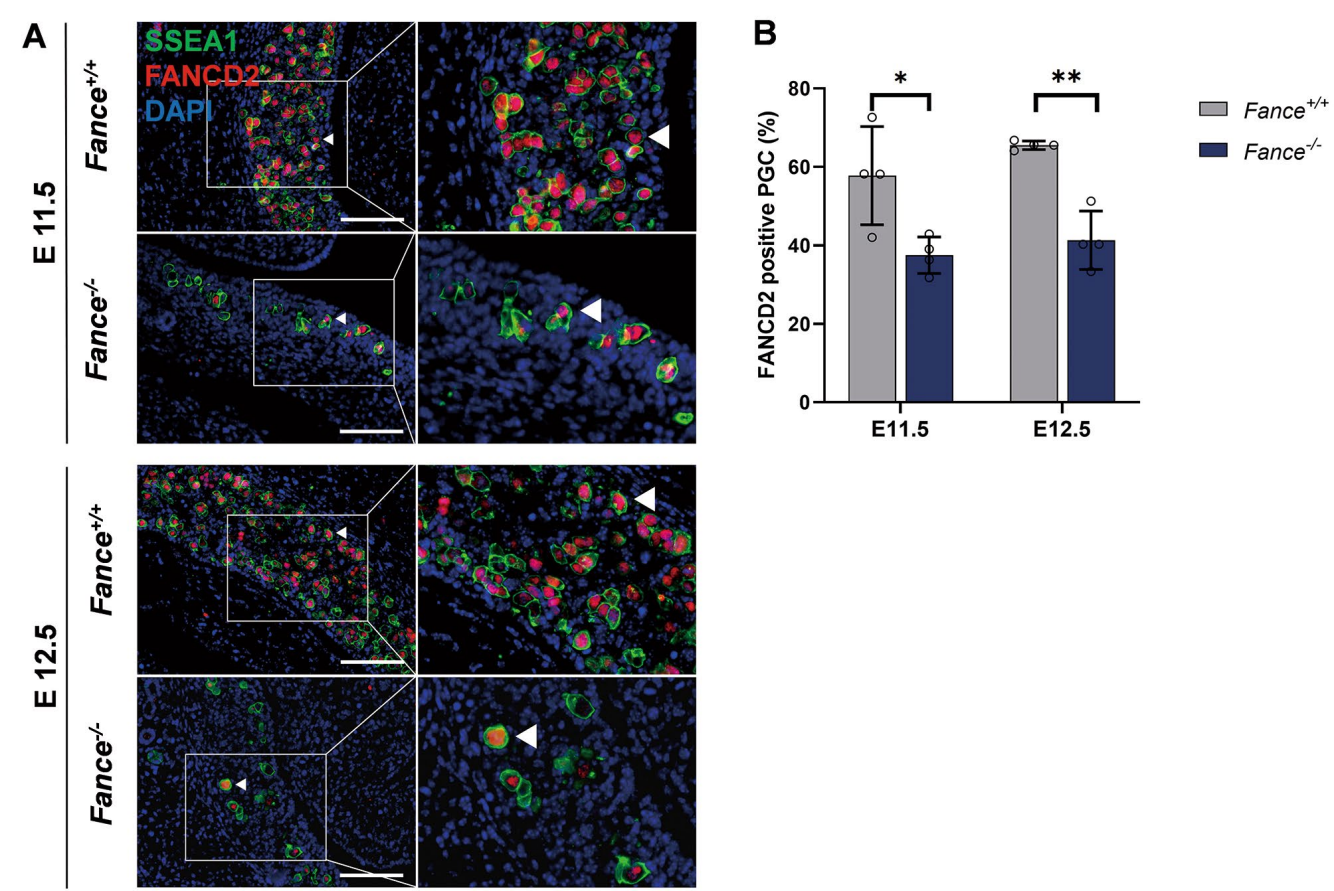
*Fance* deficient PGCs down-regulated the FA pathway during rapid mitosis. **(A)** Representative images of co-localized staining of FANCD2 and SSEA1 in *Fance*^*−/−*^ PGCs and *Fance*^*+/+*^ PGCs. **(B)** The proportion of FANCD2 positive cells was significantly decreased in *Fance*^*−/−*^ PGCs compared to *Fance*^*+/+*^ PGCs. n = 4 embryos. All bars = 200 μm

Faced the high level of TRCs induced endogenous replicative stress, PGCs require robust replication-coupled mechanisms to resolve frequent TRCs, maintain genomic stability and undergo rapid cell cycle progression. The FA pathway is involved in resolving TRCs, promoting DNA replication, and maintaining genomic stability [30–32]. RNA-Seq results showed significant decrease in RNA expression of *Blm, Rad51c, Rpa2, Brca2*, *Fance* in *Fance*^*−/−*^ PGCs at E12.5, these genes are involved in FA pathway (Fig. 4). FANCD2 serves as the focus of the FA pathway and plays a critical role in a variety of cellular activities, especially in the DNA damage response [33]. We detected significantly decreased FANCD2 expression in *Fance*^*−/−*^ PGCs at E11.5 and E12.5, compared to *Fance*^*+/+*^ PGCs (Fig. 7A-B). The results show that *Fance* deficiency causes downregulation of the FA pathway in PGCs during rapid mitosis, possibly leading to DNA repair defects and genomic instability in PGCs.

### ***Fance***^***−/−***^**PGCs down-regulated HR pathway**

**Fig. 8:**
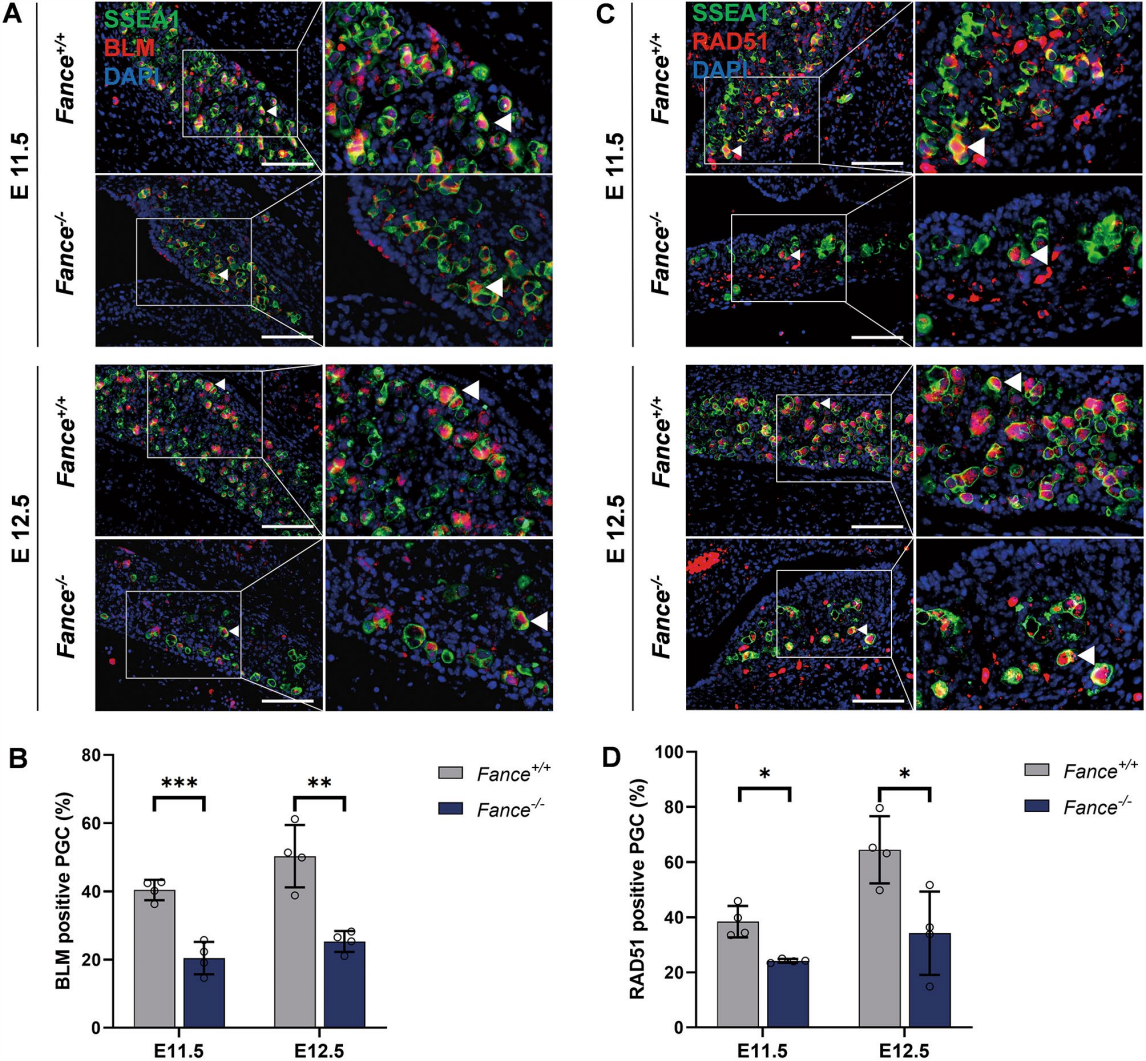
*Fance* deficient PGCs down-regulated the HR pathway during rapid mitosis. **(A)** Representative images of co-localized staining of BLM and SSEA1 in *Fance*^*−/−*^ PGCs and *Fance*^*+/+*^ PGCs. **(B)** The proportion of BLM positive cells was significantly decreased in *Fance*^*−/−*^ PGCs compared to *Fance*^*+/+*^ PGCs. **(C)** Representative images of co-localized staining of RAD51 and SSEA1 in *Fance*^*−/−*^ PGCs and *Fance*^*+/+*^ PGCs. **(D)** The proportion of RAD51 positive cells was significantly decreased in *Fance*^*−/−*^ PGCs compared to *Fance*^*+/+*^ PGCs. n = 4 embryos. All bars = 200 μm

HR and FA proteins work together to target TRCs sites in mitotic cells to prevent them from escalating into genomic instability, which is essential for cell survival and facilitates DNA replication process [34, 35]. RNA-Seq results showed significant decrease in RNA expression of *Blm, Rad51c, Rad50, Rpa2, Brca2, Pold1* in *Fance*^*−/−*^ PGCs at E12.5, these genes were involved in HR pathway (Fig. 4). We confirmed that at E11.5 and E12.5, the expression of HR pathway proteins BLM and RAD51 was significantly reduced in *Fance*^*−/−*^ PGCs, compared to *Fance*^*+/+*^ PGCs (Fig. 8A-D). In addition, BLM and RAD51 proteins are both involved in the FA pathway and the HR pathway. The results suggest that the *Fance* defect causes PGC to downregulate the HR pathway during rapid mitosis, which may contribute to DNA repair defects and DNA replication arrest in PGCs.

### ***Fance***^***−/−***^**PGCs down-regulated BER pathway**

**Fig. 9:**
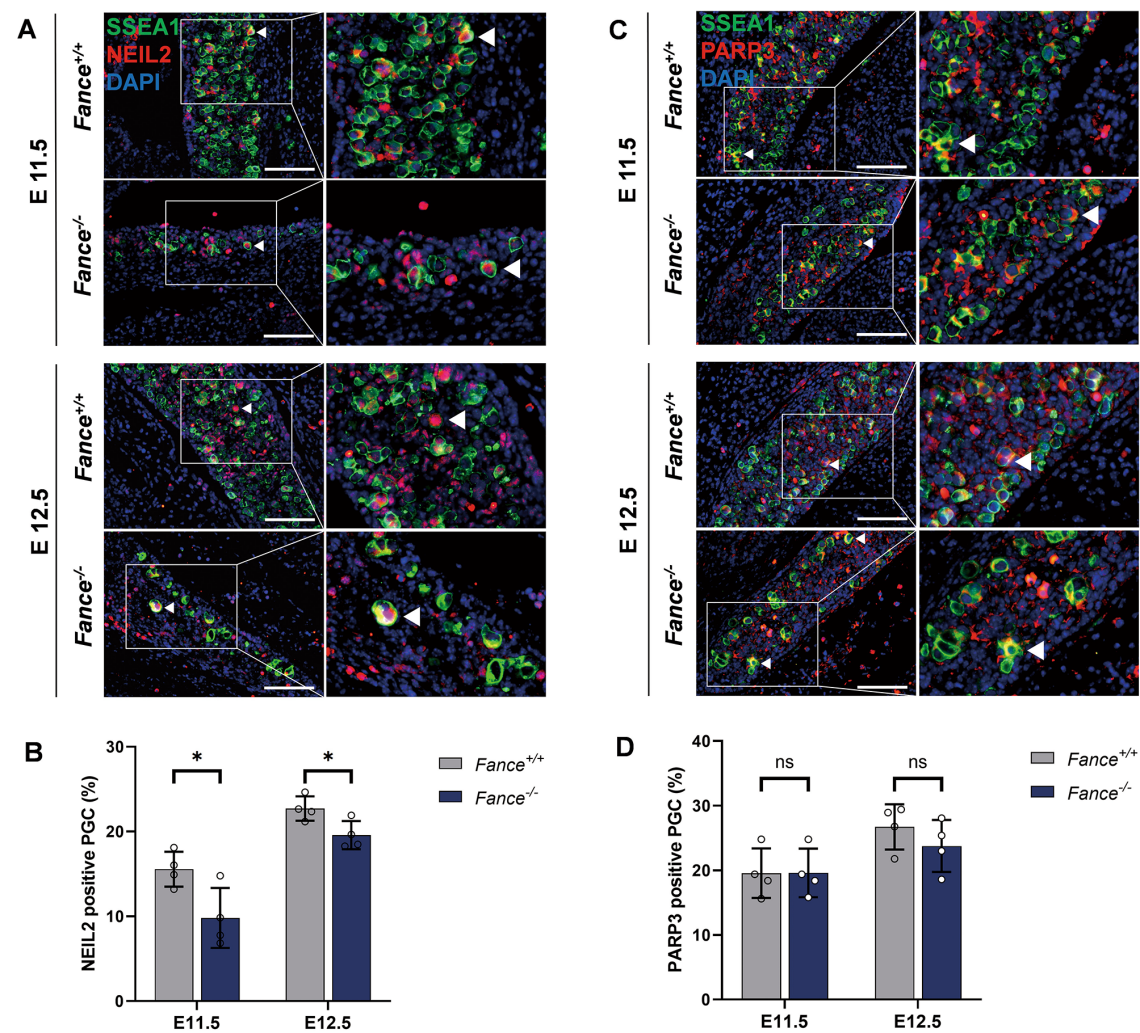
*Fance* deficient PGCs down-regulated the BER pathway during rapid mitosis. **(A)** Representative images of co-localized staining of NEIL2 and SSEA1 in *Fance*^*−/−*^ PGCs and *Fance*^*+/+*^ PGCs. **(B)** The proportion of NEIL2 positive cells was significantly decreased in *Fance*^*−/−*^ PGCs compared to *Fance*^*+/+*^ PGCs. **(C)** Representative images of co-localized staining of PARP3 and SSEA1 in *Fance*^*−/−*^ PGCs and *Fance*^*+/+*^ PGCs. **(D)** There was no significant difference in the proportion of PARP3 positive PGCs between *Fance*^*−/−*^ and *Fance*^*+/+*^ mice at E11.5 and E12.5. n = 4 embryos. All bars = 200 μm

BER mainly acts to remove abnormal bases from the strands of RNA-DNA hybrids and is an essential step in maintaining replication fork progression and keeping chromosomes stable [36]. RNA-Seq results showed significant decrease in RNA expression of *Neil2, Parp3, Pold1*, *Lig1* in *Fance*^*−/−*^ PGCs at E12.5, these genes are involved in BER pathway (Fig. 3). We verified that the expression of EBR pathway protein NEIL2 was significantly reduced in *Fance*^*−/−*^ PGCs at E11.5 and E12.5, compared with *Fance*^*+/+*^ PGCs (Fig. 9A-B). However, there was no statistical difference in the expression of EBR protein PARP3 and LIG1 protein in *Fance*^*−/−*^ PGCs and *Fance*^*+/+*^ PGCs at E11.5 and E12.5 (Fig. 9C-D), among them, LIG1 protein was also involved in DNA replication (Fig. 4A-B). The results suggest that *Fance* defects lead to the downregulation of BER in PGCs during rapid mitosis, which may result in blocked DNA replication and genomic instability of PGCs.

## Discussion

*Fance*^*−/−*^ mice showed reduced numbers of PGCs, decreased ovarian reserve, and infertility. However, the mechanisms involved in *Fance* defects leading to disrupted cell proliferation in PGCs are not completely understood. We focused on investigating the role of *Fance* defects on DNA replication and repair activities in PGCs during rapid mitosis. Our results found reduced proliferation and abnormal cell cycle distribution of *Fance*^*−/−*^ PGCs during rapid mitosis. *Fance*^*−/−*^ PGCs proliferation hindrance was associated with accumulated transcriptional replication conflict and down-regulation of FA, HR, BER repair pathways.

Our finding indicated that *Fance* defects act during rapid mitotic periods in PGCs, leading to impaired cell proliferation and genomic instability. We found reduced cell number and abnormal cell cycle distribution of PGCs during rapid mitosis. In addition, rapid cell cycle progression causes strong endogenous replicative stress, resulting in genomic instability and even promoting tumorigenesis [37, 38]. Consistently, our previous study found that *Fance* heterozygous mutant resulted in increased tumorigenesis and poor prognosis in mice [39]. The results of RNA-Seq indicated that the mechanism of *Fance* defects causing PGCs mitotic proliferation disorders is related to DNA replication and repair. We further verified the changes in TRCs and DNA repair pathways in PGCs.

Our results suggest that *Fance* defects induce PGCs to accumulate TRCs thereby hindering DNA replication. Complete and accurate DNA replication is the foundation for cell proliferation and genomic stability [40]. TRCs are inevitable during active mitosis in PGCs. Our results found a significant accumulation of R loops in *Fance*^*−/−*^ PGCs, which would exacerbate TRCs and create additional barriers to DNA replication, and induce DNA breakage and other DNA damage [41]. The resolution of high TRCs in eukaryotic cells requires multiple repair pathways, which are important mechanisms to ensure genomic stability and maintain cell survival [31]. TRCs in PGCs activate the FA pathway to maintain genomic stability [22]. Studies have shown that both ubiquitin-dependent and ubiquitin-independent functions of FANCD2, the core protein of the FA repair pathway, maintain the proliferation of PGC and establish reproductive reserve by reducing TRCs [42]. Consistently, our results show that decreased FANCD2 in *Fance*^*−/−*^ PGCs leads to intracellular accumulation of TRCs and decreased proliferation, which are important factors in reduced reproductive reserve in *Fance*^*−/−*^ mice. Studies have shown that DNA repair proteins BLM and BRCA2 cooperate with FANCD2 monoubiquitination to co-locate TRCs sites to maintain genomic stability [34]. Our study also found that the expression of HR repair protein BLM in *Fance*^*−/−*^ PGCs was significantly reduced, which further promoted the accumulation of TRCs and led to cell proliferation disorders. The BER pathway was involved in the treatment of R loops by removing the bases of RNA-DNA hybrids [43]. We found that the BER pathway in *Fance*^*−/−*^ PGCs was down-regulated, which promoted the accumulation of TRCs and leads to reduced proliferation in PGCs.

The results suggest that dysfunctional DNA repair in *Fance*^*−/−*^ PGCs contributes to reduced proliferation and genomic instability. TRCs were also a source of DNA damage. Continuous R loops can induce DNA replication fork stagnation, DNA recombination, DNA breakage, and DNA mutation [41]. The replication fork recovery mechanisms used by cells are divided into several categories: DNA damage bypass, replication fork remodeling, and replication fork breakage that generates DSBs [18]. Studies have shown that unplanned R loops in cells can lead to the formation of DSBs, and DSBs can also lead to the increase of R loops [44]. DSBs can be induced endogenously by DNA replication stress and defective DSBs repair cause replication fork stalling, collapse, or breakage, resulting in genomic instability [45]. DNA repair pathways participated by multiple DNA repair proteins function cooperatively to restore DNA replication forks. It was shown that the FA and HR pathways are coordinately involved in repairing DSBs [46–48]. Our findings revealed that FA and HR repair pathways were downregulated in *Fance*^*−/−*^ PGCs and all of these could lead to replication fork stalling owing to DNA repair defects. FANCD2 co-localizes with BRCA1 and RAD51 after monubiquitination in S phase, which collaborates with FA and HR pathways to participate in DNA repair [49, 50]. Consistently, we found significantly reduced expression of FANCD2 and RAD51 in *Fance*^*−/−*^ PGCs. The proportion of cells in S phase was reduced among *Fance*^*−/−*^ PGCs, which may also further shorten the duration of DNA repair. In addition, FA gene deficient lymphocytes showed G2 phase failure [51]. Similarly, we also found that *Fance*^*−/−*^ PGCs exhibited reduced proportion of cells in G2 phase.

The mechanism of *Fance* deficiency leading to disordered PGCs development is complicated and requires further investigation. Our study focused on exploring the transcriptional level and protein level expression of DNA replication and repair molecules in PGCs of *Fance*^*−/−*^ mice tissues. However, further in vitro validation needs to be enhanced. In addition, the role of *Fance* deficiency on the cell migration phase of PGCs also needs to be further explored.

Our findings provide theoretical basis for identifying the inherited etiology and guiding potential fertility management for POI. The identification of pathogenic variation of FA gene in POI patients by next-generation sequencing (NGS) technique was beneficial to early genetic diagnosis and early fertility management. Gonadal dysfunction and decreased fertility are common clinical manifestations in patients with FA, usually manifested as POI [6]. Studies have shown that patients with biallelic pathogenic variants of *FANCA, FANCM, FANCD1, FANCU*, and patients with monoallelic pathogenic variants of *FANCA, FANCD1, FANCL* exhibited gonadal dysfunction and infertility [52]. Our findings showed that *Fance* deficient mice exhibit PGCs dysplasia and decreased fertility, which provided potential genetic variants in POI patients. In addition, assisted reproductive technology (ART) and preimplantation genetic testing for monogenic (PGT-M) can provide some information on oocyte quality, fertilization rate, and pregnancy outcome, which can benefit fertility outcomes [53, 54]. However, high failure rates of PGT-M have been reported in FA carriers [55]. Further research is needed to explore the fertility management of POI patients with FA pathogenic variants.

In summary, we found that *Fance* defects impair the rapid mitotic proliferation of PGCs in mouse embryos, leading to a sharp decrease in PGCs number and abnormal cell cycle distribution. Proliferation disorders of *Fance* defect PGCs were associated with accumulated TRCs and down-regulation of FA, HR, BER repair pathways. Our results provide an etiologic explanation for common infertility features in FA gene deficient mammals. It also provides a theoretical basis for identifying the genetic etiology and guiding the development of potential fertility management of POI.

### Electronic supplementary material

Below is the link to the electronic supplementary material.

**Additional File 1**: **Supplementary Fig. 1***Sry* and *Fance* genotype identification in embryonic mice. Male mice show 266 bp *Sry* amplification bands as in samples 1, 2, 3, 6. Female mice do not show 266 bp bands as in samples 4, 5, 7. *Fance*^*+/+*^ mice show 620 bp as in samples 2, 6. *Fance*^*+/−*^ mice show 360 bp, 460 bp, 620 bp bands as in samples 1, 3, 4, 5. *Fance*^*−/−*^ mice show 360 bp, 460 bp bands as in sample 7.

**Additional File 2**: Supplementary Table 1 Primers used in the PCR reaction.

## Data Availability

Data and material are available upon request from the authors.

## Abbreviations

FA: Fanconi anemia
POI: Premature ovarian insufficiency
PGCs: Primordial germ cells
E: Embryonic day
TRCs: Transcription–replication conflicts
HR: Homologous recombination
BER: Base excision repair
FANCE: Fanconi anemia complementation group E
ICLs: Interstrand cross-links
DSBs: Double-strand breaks
GO: Gene Ontology
KEGG: Kyoto Encyclopedia of Genes and Genomes
DEG: Differentially expressed gene
FACS: Fluorescence activated Cell Sorting
NGS: Next-generation sequencing
ART: Assisted reproductive technology
PGT-M: Preimplantation genetic testing for monogenic
