# Supplementary material for: Fance deficiency inhibits primordial germ cell proliferation associated with transcription–replication conflicts accumulate and DNA repair defects
Source: J Ovarian Res. 2023 Aug 10;16:160. doi: 10.1186/s13048-023-01252-9 (PMC10416540; doi:10.1186/s13048-023-01252-9)
Supplement: Supplementary file 1 — Additional File 1: Supplementary Fig. 1Sry and Fance genotype identification in embryonic mice. Male mice show 266 bp Sry amplification bands as in samples 1, 2, 3, 6. Female mice do not show 266 bp bands as in samples 4, 5, 7. Fance+/+ mice show 620 bp as in samples 2, 6. Fance+/− mice show 360 bp, 460 bp, 620 bp bands as in samples 1, 3, 4, 5. Fance−/− mice show 360 bp, 460 bp bands as in sample 7. [file 13048_2023_1252_MOESM1_ESM.docx]

**Supplementary table 1** Primers used in the PCR reaction

| Gene | Sequence（5’-3’） |
| --- | --- |
| *Sry* | GAGAGCATGGAGGGCCAT  CCACTCCTCTGTGACACT |
| *Fance* | TGGCATCTCCACTTCTCTATCA  AGAGCAGCCTGGACTACTTGAG  CCTGGTGTGTAGCTTTGCCAATCA  CGTCTGTTGTGTGACTCTGGTAAC |
