# Supplementary figures and images for: Fance deficiency inhibits primordial germ cell proliferation associated with transcription–replication conflicts accumulate and DNA repair defects
Source: J Ovarian Res. 2023 Aug 10;16:160. doi: 10.1186/s13048-023-01252-9 (PMC10416540; doi:10.1186/s13048-023-01252-9)

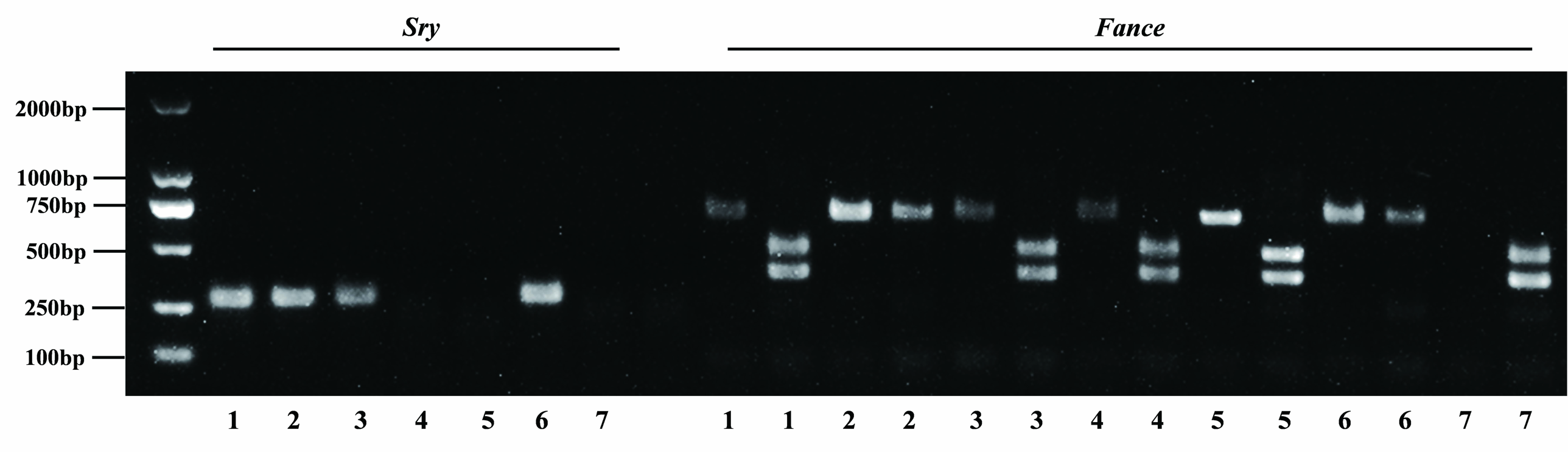

Supplement: Supplementary file 2 — Additional File 2: Supplementary Table 1 Primers used in the PCR reaction. [file 13048_2023_1252_MOESM2_ESM.jpg]
